# Ground Reaction Force Estimates from ActiGraph GT3X+ Hip Accelerations

**DOI:** 10.1371/journal.pone.0099023

**Published:** 2014-06-10

**Authors:** Jennifer M. Neugebauer, Kelsey H. Collins, David A. Hawkins

**Affiliations:** 1 Biomedical Engineering Graduate Group, University of California Davis, Davis, California, United States of America; 2 Department of Neurobiology, Physiology & Behavior, University of California Davis, Davis, California, United States of America; The University of Queensland, Australia

## Abstract

Simple methods to quantify ground reaction forces (GRFs) outside a laboratory setting are needed to understand daily loading sustained by the body. Here, we present methods to estimate peak vertical GRF (pGRFvert) and peak braking GRF (pGRFbrake) in adults using raw hip activity monitor (AM) acceleration data. The purpose of this study was to develop a statistically based model to estimate pGRFvert and pGRFbrake during walking and running from ActiGraph GT3X+ AM acceleration data. 19 males and 20 females (age 21.2±1.3 years, height 1.73±0.12 m, mass 67.6±11.5 kg) wore an ActiGraph GT3X+ AM over their right hip. Six walking and six running trials (0.95–2.19 and 2.20–4.10 m/s, respectively) were completed. Average of the peak vertical and anterior/posterior AM acceleration (ACCvert and ACCbrake, respectively) and pGRFvert and pGRFbrake during the stance phase of gait were determined. Thirty randomly selected subjects served as the training dataset to develop generalized equations to predict pGRFvert and pGRFbrake. Using a holdout approach, the remaining 9 subjects were used to test the accuracy of the models. Generalized equations to predict pGRFvert and pGRFbrake included ACCvert and ACCbrake, respectively, mass, type of locomotion (walk or run), and type of locomotion acceleration interaction. The average absolute percent differences between actual and predicted pGRFvert and pGRFbrake were 8.3% and 17.8%, respectively, when the models were applied to the test dataset. Repeated measures generalized regression equations were developed to predict pGRFvert and pGRFbrake from ActiGraph GT3X+ AM acceleration for young adults walking and running. These equations provide a means to estimate GRFs without a force plate.

## Introduction

Ground reaction forces (GRFs) are of interest for many applications, such as quantifying loads sustained by the body during various activities of daily living. Currently however, quantifying GRFs is typically limited to a laboratory setting and therefore may not accurately reflect the loading sustained during daily living. Profiles of the forces sustained during daily living could provide insights into pre- and post-surgical outcomes [1], correlations with bone density [2]–[4], loading sustained by populations at risk for bone loss [5], and provide critical data for developing injury prevention interventions [6]–[8]. The development of a simple, portable, and inexpensive method to quantify GRFs during daily living must be identified.

Accelerometry-based activity monitors (AMs) are small devices most often worn on a person's hip or wrist to quantify physical activity. AMs are most commonly used to estimate energy expenditure during various tasks [9]–[14]. More recently, both the AM acceleration and AM counts, which are calculated from acceleration [15], have been related to GRFs during walking and running [16]–[19]. To date, a regression equation to estimate peak vertical GRF (pGRFvert) in adults, similar to that developed for girls and boys (ages 10–14) [18] has not been identified. Furthermore, a similar equation for braking GRFs (pGRFbrake) has not been developed. With recent developments in AM technology, raw triaxial accelerations collected at 100 Hertz (Hz) (compared to 40 Hz sampling rate used by Neugebauer, et al. (2012)) provide the opportunity to develop equations to predict both pGRFvert and pGRFbrake. While the AMs could be used as designed to simply quantify accelerations during locomotion, the ability to quantify both accelerations and GRFs outside of a laboratory provides additional information currently not available to biomechanists and/or clinicians. GRFs are the external forces applied to the body and therefore provide fundamental information about the mechanical loading sustained by the body. Simply reporting accelerations, as measured by the AMs, does not provide this same information. Vertical and braking GRFs are the two largest components of GRFs during locomotion. Each provides unique information that if combined to examine resultant GRF would be lost. Anterior/posterior forces dictate locomotion speed [20] and may play an important role in knee ligament loading/injury. Estimating these force components individually may be of interest to some investigators and therefore two equations (i.e. one for peak vertical force and one for peak braking forces in the anterior/posterior direction) were developed rather than one equation to estimate resultant GRF.

The ActiGraph GT3X+ AM is a triaxial accelerometer capable of sampling up to 100 Hz. The GT3X+ provides access to the raw triaxial acceleration data, allowing for analysis of accelerations not only in the vertical direction but also in the anterior/posterior direction. This allows the development of regression equations to predict pGRFvert as well as pGRFbrake. The purpose of this study was to develop generalized equations using raw hip acceleration, as measured by the ActiGraph GT3X+ AM, to predict pGRFvert and pGRFbrake during walking and running in a young adult population.

## Methods

### Ethics Statement

This study was approved by the University of California, Davis Institutional Review Board and prior to testing, all subject gave written informed consent.

### Participants

44 subjects (23 male and 21 female) participated in this study. Subjects were free of any lower extremity pain or injury in the six months prior to participating in the study. For each subject, body mass and height were measured to the nearest 0.1 kg and 0.5 cm, respectively. Body mass index (BMI (kg/m**^2^**)) was calculated for all subjects.

### Protocol

Each subject wore an ActiGraph GT3X+ AM (range ±6 g with a sampling rate 100 Hz; ActiGraph, Pensacola, FL) secured on a nylon belt around their waist and located over the most lateral aspect of their waist (i.e. over the right iliac crest). Subjects were briefed on the protocol and practiced walking and running until both the investigator and subject felt they were prepared to successfully complete the various gait trials. Trials were considered successful if the right foot fully contacted the force plate without any apparent gait alteration.

Subjects completed an average of eight to ten walking (speeds of 0.95–2.19 m/s at 0.2 m/s increments) and eight to ten running (speeds of 2.20–4.10 m/s at 0.3 m/s increments) trials (the order of walk and run trials was randomly assigned) along a 15 m straight path, which included a force plate (Kistler Corporation, Model 9281B (40×60 cm), Amherst, NY, USA) about 6 m from the starting point. Locomotion speed was determined using electronic timing gates located two meters on either side of the force plate and synchronized with force plate data acquisition. Subjects were initially asked to walk and run at self-selected speeds. After each self-selected speed trial, subjects were instructed to ‘speed up a little’ or ‘slow down a little’ to obtain additional speeds within 0.2 or 0.3 m/s increments for walking and running, respectively. Force plate data were collected using a custom Labview (National Instruments Corporation, Austin, TX) data acquisition program, sampled at 1000 Hz. The AM and data collection computer were synchronized to an atomic clock that was used to identify the start time of each trial.

The pGRFvert and pGRFbrake (Newtons (N)) for a single step within each trial were determined from unfiltered GRF data using a custom LabView program. The specific peak AM acceleration that corresponded with contact to the force plate could not be identified (due to a limitation in the ability to synchronize the AM and force plate data collection), and therefore the average of the peak vertical and peak braking AM accelerations (ACCvert and ACCbrake, respectively; 1 g = 9.807 m/s^2^) over 10 seconds after the start of the trial were determined for each trial using a custom LabView program (standard deviations for all trials averaged 7.8% (±3.0%) and 14.9% (±7.7%) in the vertical and anterior/posterior directions, respectively while the coefficient of variations averaged 0.08 (±0.03%) and 0.15% (±0.08%) in the vertical and anterior/posterior directions, respectively). Hip accelerations were taken as reported by ActiGraph without additional signal processing.

For each subject, six walking and six running trials were selected for inclusion in the final dataset. Each of the six trials selected were completed at a unique speed over the range tested. For 5 of the 44 subjects (∼11%; 4 males and 1 female), average ACCvert was 6 g (indicating saturation of the accelerometer's 6 g maximum) for the majority of the running trials. These subjects were therefore excluded from all data analyses due to signal saturation.

### Statistical Analysis

Means and standard deviations were determined for subject demographics. Thirty subjects were randomly assigned to the training dataset, used to develop the models, leaving the remaining 9 assigned to the test dataset to be used to cross validate the models using a holdout approach [21], [22]. Repeated measures generalized models were developed for pGRFvert and pGRFbrake in R (R Foundation for Statistical Computing, Austria) using the training dataset. The factors included in the models were selected after careful consideration of basic mechanics principles and experience from development of similar models for children [18]. Equations of motion that describe leg segment dynamics during gait were derived and arranged to express GRF as a function of hip acceleration and all other relevant quantities including limb segment lengths, center of mass locations, masses, moments of inertia, angular velocity, and linear and angular accelerations. For a given subject, several quantities in this relationship would remain constant such as segment lengths, center of mass locations, masses and moments of inertia. Other quantities such as segment angular velocities, linear and angular accelerations are directly related to locomotion speed, type of gait, and movement mechanics, which can vary between sexes and individuals. It would be difficult and time consuming to try and collect all these quantities for a given person and would limit the utility and convenience of a GRF prediction model. Fortunately, we determined from previous studies of children [18] that by including gross body mass and height (to account for anthropometric quantities), and hip acceleration, sex, subject and gait type (to account for individual gait characteristics) in mixed and generalized peak vertical GRF (pVGRF) prediction models, we were able to predict pVGRF within 5% and 9% of actual values respectively. Therefore, in this study, we considered similar factors in the model. The models initially included the fixed effects of ACCvert or ACCbrake (ACC in similar direction as the force being predicted), sex (where male  = 0 and female  = 1), height, mass, type of locomotion (where walk  = 0 and run  = 1), and the interaction of type of locomotion and AM acceleration. Subject was included as a random effect to account for the repeated measures. Significance of p <0.05 was used to determine if a variable remained in the equation. These models were powered at 0.80 [23].

The coefficients derived for each model were used to create equations to predict pGRFvert and pGRFbrake. The equations were then used to predict pGRFs for subjects in the test dataset. The predicted pGRFs for each subject were compared to the measured pGRFs for both the vertical and braking directions and average absolute percent difference calculated for each subject. The average and standard deviation of the average absolute percent difference for all subjects within the test dataset were determined. Model assumptions (linearity of relationships, normality and homoscedasticity of residuals) were checked via residual analyses (Q-Q plots, and summary diagnostics) to ensure that both the prediction equations and the single-number summaries of the predictions accurately represent the full dataset. Using the test dataset only, bias of the models is reported as the mean of the difference between the actual and model predicted GRFs. Upper and lower agreement limits were determined using ±2 standard deviations of the mean of the difference between the actual and the model predicted pGRFs for the test dataset.

## Results

The general characteristics of the subjects tested were unremarkable (Table 1). There were no differences between sexes in age and BMI. There were significant differences between sexes in height (p<.05) and body mass (p<.0001), There were no significant differences between “training” and “test” subjects within sex.

**Table 1:** Subject demographics for study population.

	Males		Females	
	Training	Test	Training	Test
n	15	4	15	5
**Age (years)**	20.9±1.5	21.3±1.3	21.3±1.1	21.8±0.8
**Height (m)**	1.82±0.07	1.78±0.16	1.65±0.09 *	1.68±0.11 *
**Mass (kg)**	74.1±9.6	74.0±15.0	60.4±8.1 *	64.9±11.9 *
**BMI (kg/m^2^)**	22.5±2.7	23.1±1.1	22.3±2.1	22.8±1.8

Mean ± one standard deviation are reported.

* Significant (p<0.05) difference between males and females.

### Vertical Ground Reaction Force

**Figure 1:**
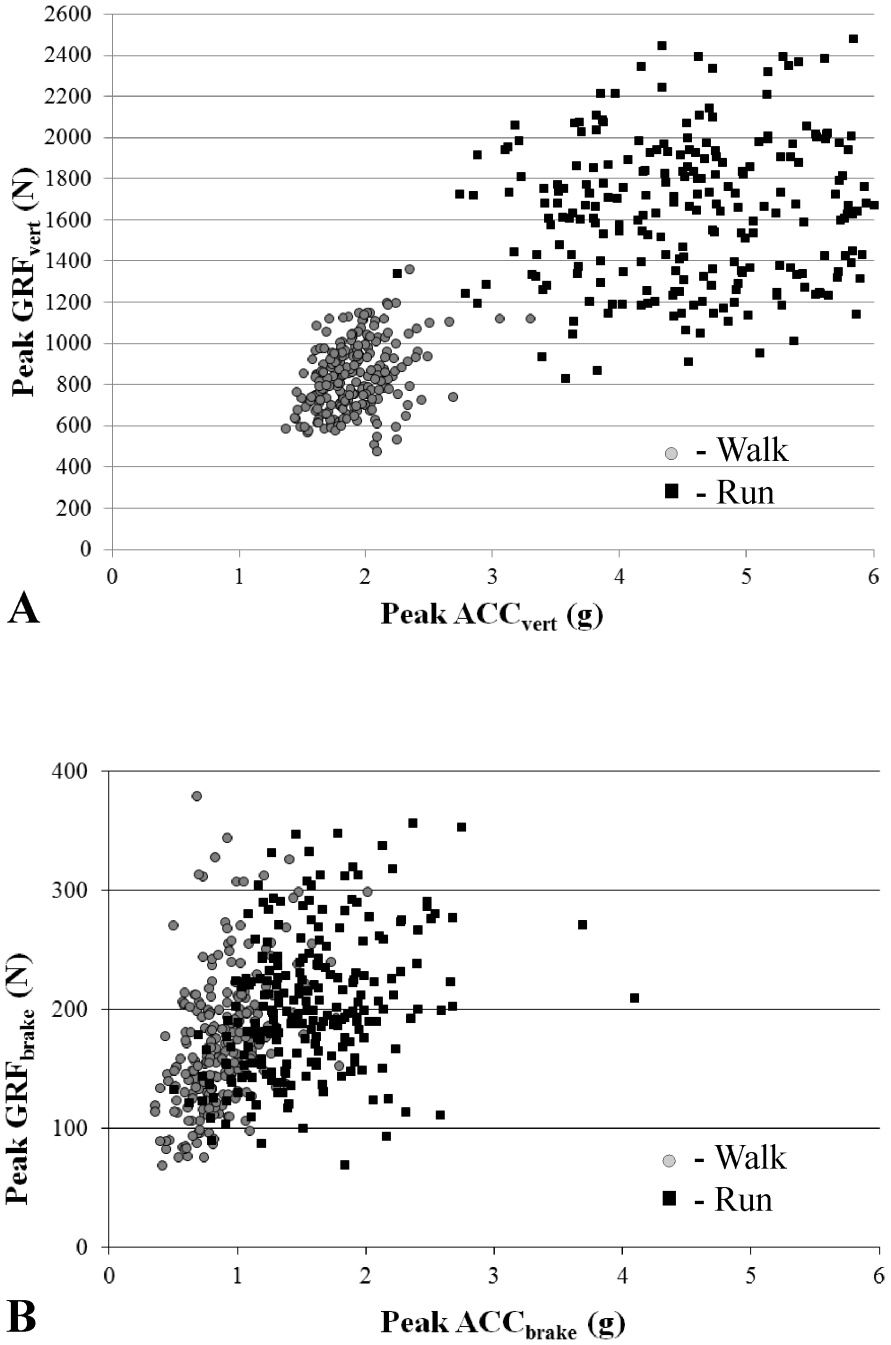
Scatter plot of pGRFvert (A) and pGRFbrake (B) versus respective average of peak ACC for all trials. Walking trials are shown in gray circles and running trials in black squares.

**Figure 2:**
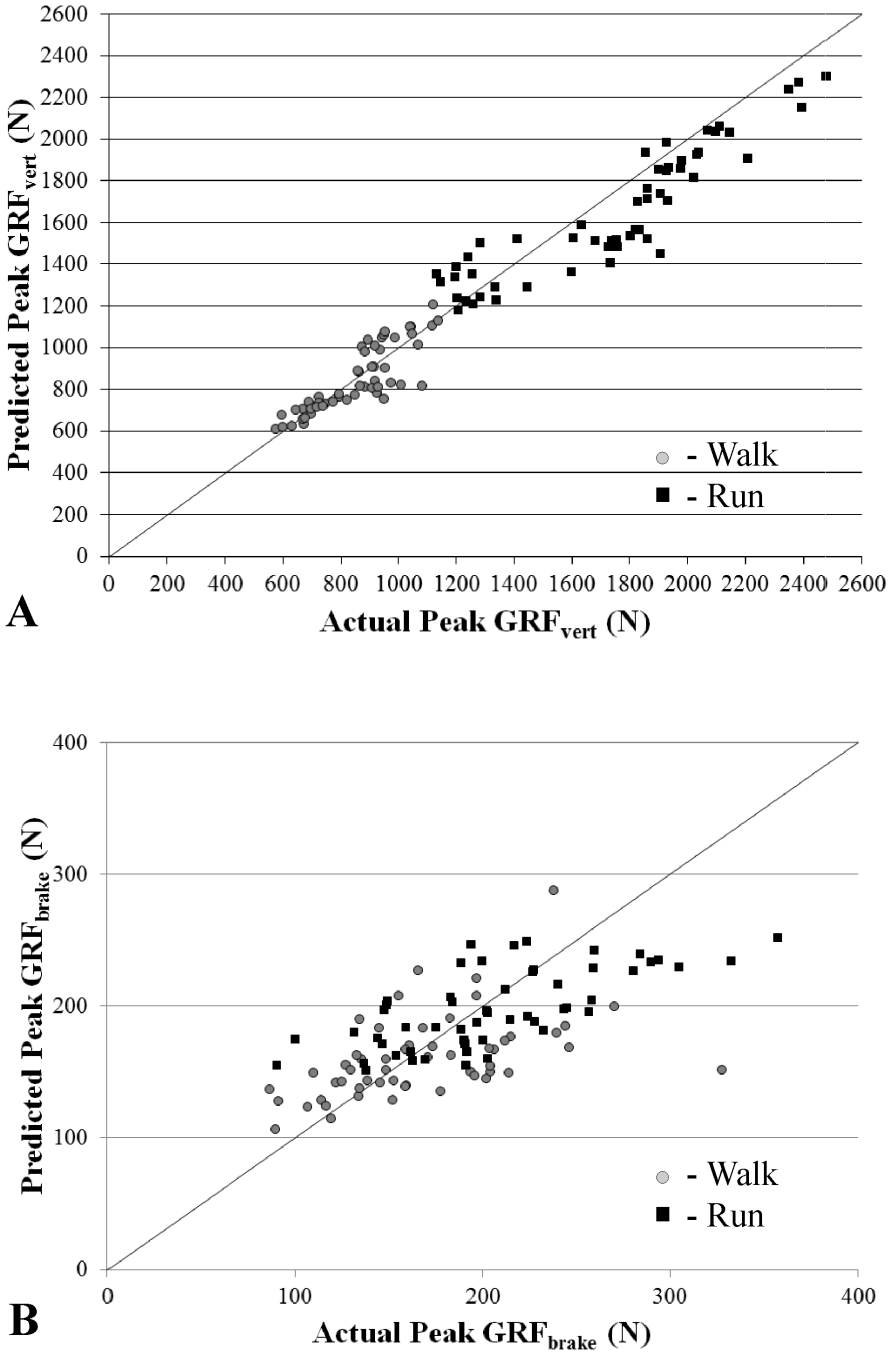
Predicted versus actual pGRFvert (A) and pGRFbrake (B) using the generalized models applied to subjects in the test dataset. The actual versus predicted fit for pGRFvert and pGRFbrake generalized models resulted in an r^2^ = 0.94 (p<0.001) and r^2^ = 0.43 (p<0.001), respectively. Walking trials are shown in gray circles and running trials in black squares.

A generalized equation to predict pGRFvert was developed that included four significant factors. ACCvert increased as pGRFvert increased during walking and running trials (Figure 1A). Natural log transformation of pGRFvert was used to account for the non-Gaussian distribution. Significant factors in the generalized pGRFvert model (Table 2) included ACCvert, mass, type of locomotion, and the interaction between ACCvert and type of locomotion (Equation 1). A one g increase in ACCvert represents a 31.1% and a 5.7% increase in GRFvert for walking and running, respectively. Each added kg of mass was associated with 1.4% increase in pGRFvert. The average absolute difference between actual and predicted pGRFvert was 8.3±3.7% (106.4 N) for subjects in the test datasets (Figure 2A).

**Table 2:** Coefficients for the pGRFvert and pGRFbrake generalized models.

	Subscript	Vertical	Braking
		α	ω
		Equation 1	Equation 2
Intercept	0	5.247	3.773
ACC (g)	1	0.271	0.665
Mass (kg)	2	0.014	0.011
Type of locomotion (walk/run where walk = 0 and run = 1)	3	0.934	0.505
ACC*run interaction	4	−0.216	−0.491

All factors were significant (p<0.001).



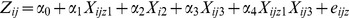
(1)where Z_ij_  =  log-transformed pGRFvert (ln(N)) for subject i, trial j

X_ijz1_  =  ACCvert (g)

X_i2_  =  mass (kg)

X_ij3_  =  type of locomotion (where walk  = 0 and run  = 1)

α  =  coefficient associated with respective variable

e_ijz_  =  error in trial j for subject i for direction z (vertical)

**Figure 3:**
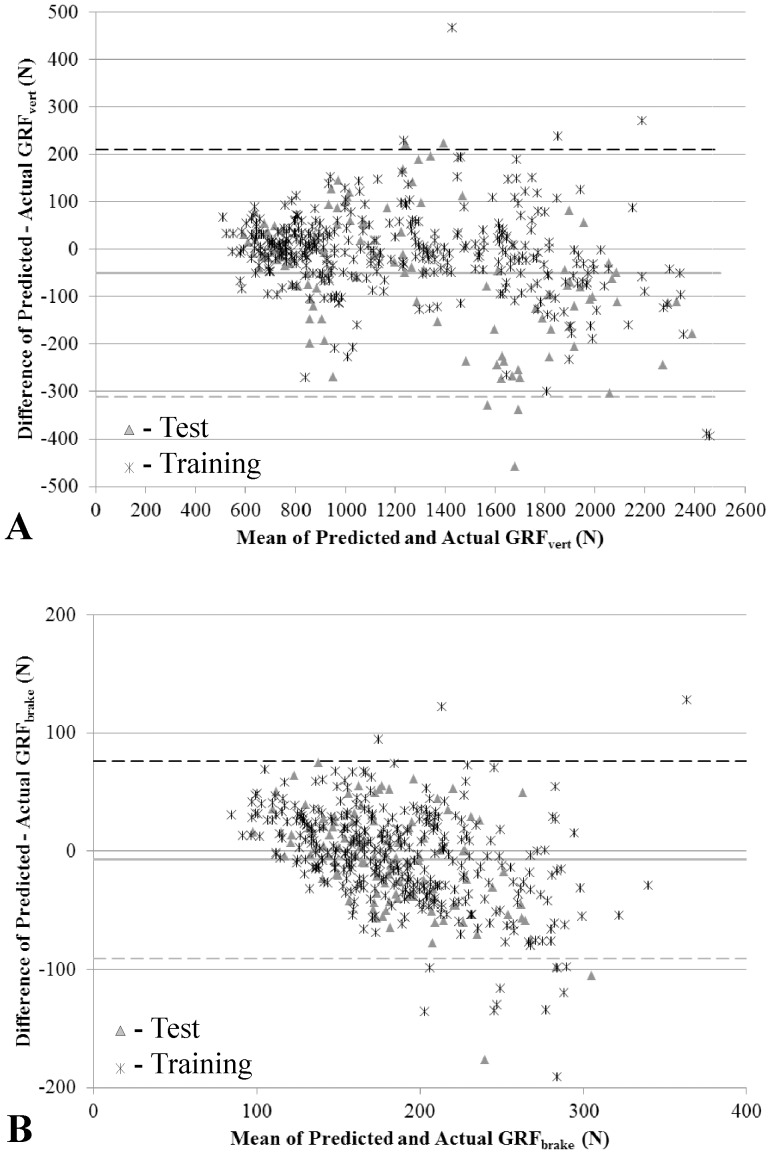
Bland Altman plots for pGRF_vert_ (A) and pGRF_brake_ (B) for subjects in both the test (triangles) and training datasets (stars). Upper (black dashed line) and lower (gray dashed line) agreement limits and the bias (gray solid line) were calculated using the test dataset only.

Bland-Altman upper and lower limits of agreement are 210.3 N and −311.3 N, respectively (Figure 3A). The mean bias ±1 standard deviation is −50.5±130.4 N, suggesting that the model underestimates pGRFvert.

### Braking Ground Reaction Force

A generalized equation to predict pGRFbrake was developed that included four significant factors. ACCbrake increased as pGRFbrake increased during walking and running (Figure 1B). Natural log transformation of pGRFbrake was used to account for the non-Gaussian distribution. Significant factors in the generalized pGRFbrake model included ACCbrake, mass, type of locomotion, and the interaction between ACCbrake and type of locomotion (Equation 2). A one g increase in ACCbrake represents a 94.5% increase and a 19.0% increase in GRFbrake for walking and running, respectively (Table 2). The average absolute difference between actual and predicted pGRFbrake was 17.8±4.0% (33.2 N) for subjects in the test dataset (Figure 2B).
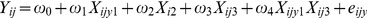
(2)where Y_ij_  =  log-transformed pGRFbrake (ln(N)) for subject i, trial j

X_ijy1_  =  ACCbrake (g)

X_i2_  =  mass (kg)

X_ij3_  =  type of locomotion (where walk  = 0 and run  = 1)

ω  =  coefficient associated with respective variable

e_ijy_  =  error in trial j for subject i for direction y (braking)

Bland-Altman upper and lower limits of agreement are 76.4 N and −91.0 N, respectively (Figure 3B). The mean bias ±1 standard deviation is −7.3±41.9 N, suggesting that the model underestimates pGRFbrake.

## Discussion

Generalized regression equations were developed to estimate pGRFvert and pGRFbrake in young adults using hip acceleration measured with an AM. AMs have been used and validated to estimate energy expenditure [9], [24] but have received less attention for the relationship between GRFs and AM accelerations [16]–[19]. Previous work developed a statistically based regression equation to estimate pGRFvert that was limited to (1) a youth population, (2) 15 second epochs of average resultant acceleration, and (3) 40 Hz sampling rate [18]. With the availability of raw triaxial accelerations, equations to estimate both pGRFvert and pGRFbrake are possible. Estimates of peak GRFs based on raw accelerations (rather than epochs) eliminate the potential for underestimating peak forces due to averaging that occurs with epochs [19]. We focused on GRFs rather than simply quantifying accelerations because GRFs are commonly measured with most biomechanical evaluations and provide a means to estimate total loading of the body. Accelerations are typically measured for a specific segment and attenuate as you move from the foot towards the head [25], [26]. Because of this attenuation, accelerations from a hip mounted AM may not provide an accurate representation of the load sustained by the body. Therefore, the aim of the present study was to develop generalized equations using an ActiGraph GT3X+ AM, an AM capable of reporting raw triaxial acceleration, to predict pGRFvert and pGRFbrake during walking and running in an adult population.

Using the generalized model to predict peak GRFvert resulted in an average absolute percent error of approximately 8%, similar to previously reported percent errors [18], with a bias of −50.5 N (Figure 3) suggesting that the model underestimates. Additional research is need to determine if developing a model using the peak hip ACC that corresponds to the step that the GRF was measured would reduce the percent error and the bias. The significant factors (acceleration, mass, type of locomotion, and the interaction between acceleration and the type of locomotion) for predicting pGRFvert were similar to those previously reported even though this study used a different AM, raw acceleration, and an older population [18].

In addition to a predictive equation for pGRFvert, an equation to predict pGRFbrake was developed. Mass, type of locomotion, and the interaction between type of locomotion and ACCbrake were significant factors, similar to the pGRFvert equation. pGRFbrake was less well predicted (average absolute percent error ∼18%) than pGRFvert with a bias of −7.3 N. Previous studies have not predicted pGRFbrake using AM acceleration. One variable thought to possibly affect the prediction was speed. GRFs generally increase with increasing speed [27], [28]. With speed included as a fixed effect in the generalized model, the average absolute error decreased with a slight increase in standard deviation (15.6±6.2% or 29.3 N). We did not include locomotion speed in the final model because it is more challenging to determine outside of a laboratory. For pGRFbrake, additional investigation into significant factors is needed to decrease the prediction errors. Factors added to the model should be easily quantified in the field and/or could be assumed to be constant for a subject, such as foot strike pattern [29]–[31] that might be detectable based on acceleration profiles.

Of note in this study is the significance of type of locomotion in predicting both pGRFvert and pGRFbrake. Type of locomotion and an interaction between type of locomotion and ACC were significant in both equations, consistent with previous work [18]. Running was associated with significantly greater peak GRFs as ACC increased. While previous studies have considered walking and running [17], [19], [24], as little as one walking and one running speed have been used to characterize each. Results from this study as well as from a previous study [18], [24] consistently demonstrate that multiple walking and running speeds should be included in an equation that relates pGRFs to ACC. Simply including one walking and running speed to characterize each type of locomotion does not fully describe the relationship between the accelerations and forces sustained.

Estimating GRFs during daily living may be highly relevant to the investigation of GRFs and bone health and bone mineral density [2]–[6]. Bone is known to remodel in response to the loading sustained. The development of models to estimate GRFs using an AM provide a means for researchers to estimate GRFs during daily activities over multiple days/weeks. Loading profiles could then be related to bone health. GRFs are the external loads applied to the lower extremity during gait and thus provide a direct mechanical stimulus to bone. Previous studies have identified associations between GRFs and bone mineral density [2] that could be further explored in a larger study such as the Iowa Bone Development Study [17] or the National Health and Nutrition Examination Survey [32] by applying models such as those presented here. Additionally, the models presented here provide both vertical and braking GRFs rather than just summed or resultant GRFs in order to further understand bone mineral density and overall bone health.

The equations presented here could also be used by clinicians to monitor gait alterations [7] when patients are outside the clinic. Recent studies have shown biofeedback as an effective means for runners to decrease peak GRFs to decrease the risk of tibial stress fracture [8]. Adherence to this altered gait when patients are not being watched by clinicians is currently unknown. Providing an AM to a patient to wear during training runs could provide the clinician an objective means to capture and illustrate the GRFs sustained during runs and determine if gait retraining has been implemented outside the laboratory. GRFs play an important role in knee injury mechanisms and thus being able to track peak vertical and braking GRFs in-the-field may provide valuable information needed to develop injury prevention models. For example, tracking GRFs during training could be used to; [1] determine if GRFs increase during certain drills or over time, perhaps as muscles fatigue, and [2] if the incidence of knee injuries increases as GRFs increase. Such information could be used to identify an athlete's increased injury risk in real-time.

While the results of this study provide novel means to estimate GRFs during daily living, several limitations of this study should be noted. First, the equations presented here were developed with a hip worn Actigraph GT3X+. Applying these equations to AMs worn differently, such as on the wrist, should be explored before use. Accelerations, as measured by AMs, may differ at the wrist compared to those measured at the hip for the same activity [19]. Second, the equations developed here are applicable to walking and running only. The application of these equations to predict GRFs during jumping or other more ballistic tasks is unknown. Additionally, these equations require a means to determine if a given acceleration represents walking or running. Automated methods to distinguish between walking and running from inertial sensors have been reported [33]–[35] and could be implemented in combination with the equations presented here. Third, data for five subjects (11%; 4 male, 1 female) were excluded from this analysis due to saturation of the peak vertical accelerations during running. Of the excluded subjects, four were male and weighed more (84.8±13.9 kg) with comparable height (1.79±0.10 m) compared with the subjects included in the analysis. Previous studies have reported peak running accelerations greater than 6 g during daily activities [7] including running [8], [31], which are consistent with the current findings. The saturated accelerations by these 5 subjects combined with the previously reported running peak accelerations highlight the importance of progressing to AMs with greater acceleration ranges (e.g. greater than ±11 g).

The present study developed equations to predict both pGRFvert and pGRFbrake. Significant factors for both equations included AM acceleration (in the same direction as the force being predicted), mass, type of locomotion, and an interaction between AM acceleration and type of locomotion. The pGRFvert equation predicts pGRFvert with an 8% average absolute percent error. The larger average absolute percent error in predicting pGRFbrake compared with that in predicting pGRFvert suggests that additional factors could be included in the equation to improve the predictions. These equations provide the foundation for predicting GRFs during daily living outside of laboratory settings.

## Acknowledgments

The authors would like to thank the subjects for volunteering to participate in this study. Thanks to Erin Dienes for statistical programming advice.
